# Osteomyelitis Complicating Sickle Cell Disease

**DOI:** 10.5334/jbsr.3980

**Published:** 2025-08-08

**Authors:** Furgan Özcan, Filip Vanhoenacker, Frederick Catry

**Affiliations:** 1 Department of Radiology, University Hospital Brussels, VUBrussel, Belgium; 2 Department of Radiology, AZ Sint‑Maarten, Mechelen, Belgium; 3 Faculty of Medicine and Health Sciences, University of Antwerp and Gent, Belgium

**Keywords:** sickle cell disease, bone infarct, osteomyelitis, MRI

## Abstract

*Teaching point:* Differentiating diaphyseal osteomyelitis and bone infarction in patients with sickle cell disease is challenging and requires meticulous correlation of imaging and clinical findings.

## Case History

A 3‑year‑old child of African descent presented with high fever, limping, and refusal to bear weight. There was no history of trauma.

Abdominal ultrasound was unremarkable.

**Figure 1:**
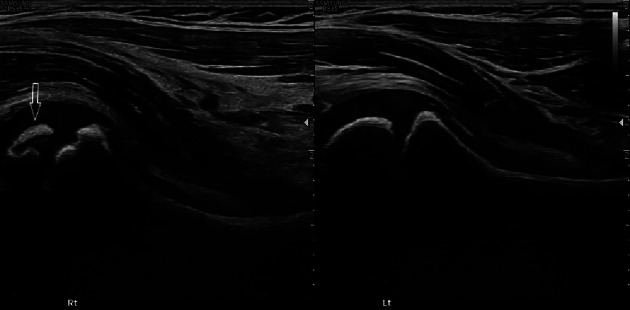
Ultrasound. Irregular delineation of the right proximal femur (arrow).

Ultrasound of the hips demonstrated irregular delineation of the right proximal femoral epiphysis and adjacent growth plate (Figure 1, arrow).

**Figure 2:**
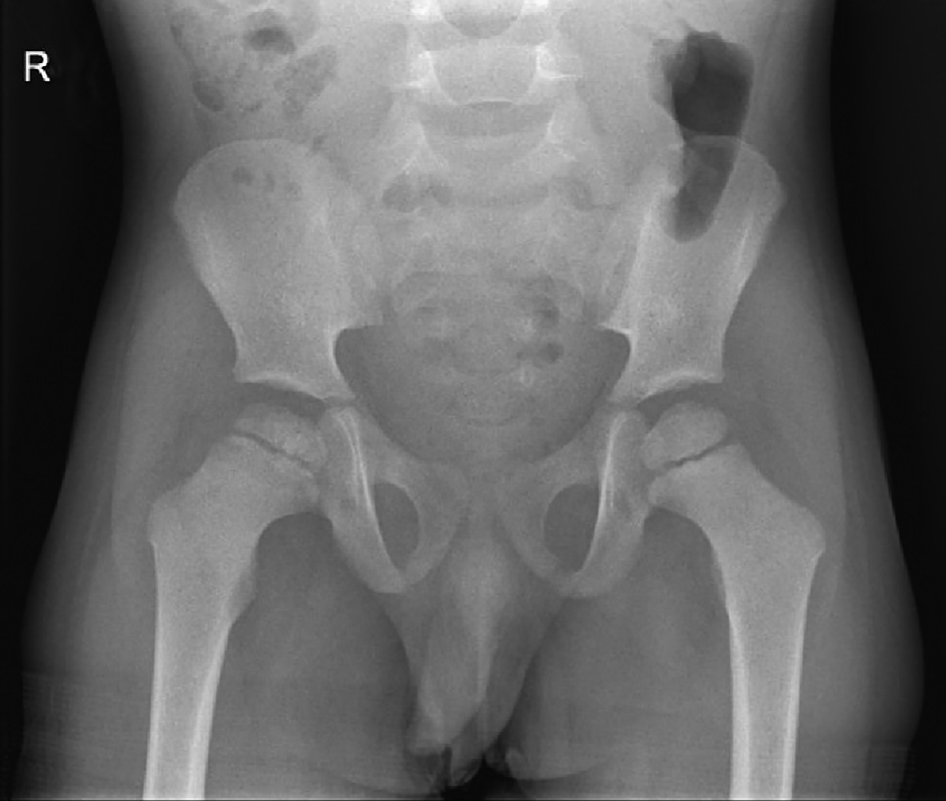
Conventional radiography. Flattening, sclerosis of femoral head; widening of femoral neck.

Conventional radiography (CR) of the pelvis showed flattening, heterogeneous sclerosis of the right femoral head, and widening of the femoral neck (Figure 2), in keeping with Legg–Calvé–Perthes disease.

Laboratory examination revealed leukocytosis with a left shift, anemia, and elevated CRP (116 mg/L). Hemoglobin electrophoresis revealed sickle cell disease (SCD) by demonstrating an elevated HbS fraction.

**Figure 3:**
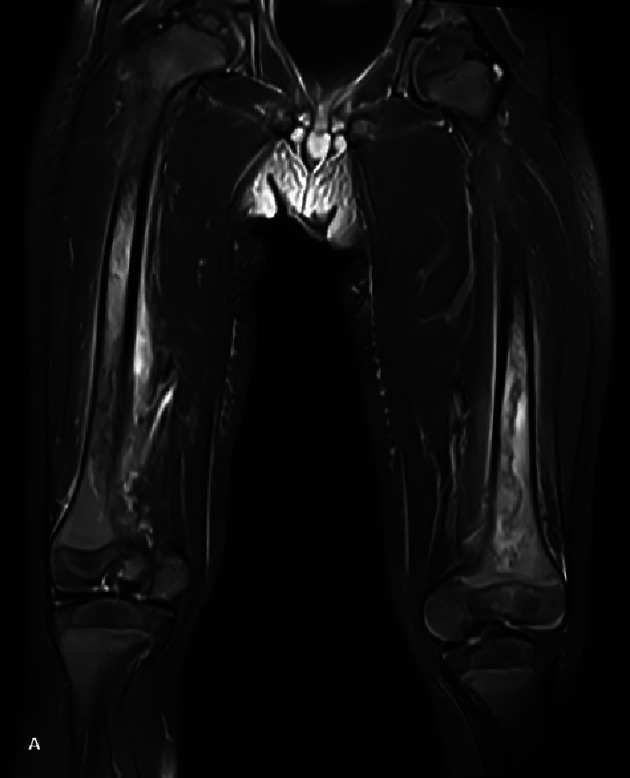

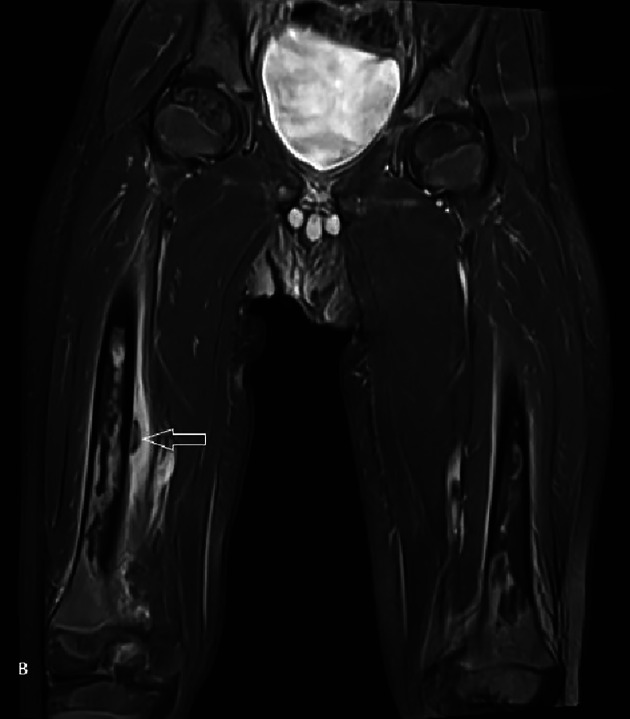

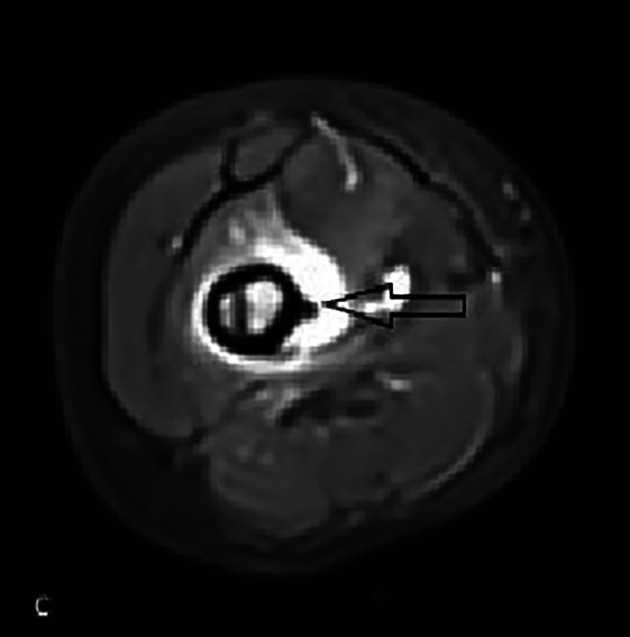
**A** Fat‑saturated PD‑WI MRI. Increased signal in the femoral diaphyses. **B–C** Fat‑saturated T1‑WI MRI with contrast. Subperiosteal collection with peripheral rim enhancement (arrow).

Because of clinical suspicion of osteomyelitis, an MRI was performed. Fat suppressed (FS) proton density (PD) image (Figure 3A) revealed diffuse increased signal intensity in both femoral diaphysis, indicating red bone marrow hyperplasia.

A subtle subperiosteal collection along the right femoral diaphysis, with peripheral rim enhancement (Figure 3B‑C, arrow) was seen on contrast‑enhanced FS‑T1‑weighted images.

The diagnosis of SCD complicated by osteomyelitis was suggested, based on the combination of clinical, laboratory, and imaging findings. Blood cultures remained negative. The patient showed uneventful recovery within two weeks following the start of antibiotic therapy, supporting empirically the diagnosis of osteomyelitis.

## Comments

SCD is an autosomal recessive disorder of the hemoglobin molecule in red blood cells, causing chronic hemolytic anemia and chronic hypoxemia that may result in compensatory red bone marrow hyperplasia.

The most common musculoskeletal (MSK) complications include avascular necrosis or bone infarct due to microvascular vaso‑occlusion, osteomyelitis (typically caused by Salmonella), septic arthritis, and growth retardation [1]. Legg–Calvé–Perthes disease—as in our patient—is the pediatric equivalent of avascular necrosis of the hip in adults.

Acute infarction and acute osteomyelitis of the diaphysis of the long bones may present with similar features on MRI [1], rendering the differential diagnosis difficult, particularly if pre‑existing bone marrow hyperplasia is present. Clinical presentation is often nonspecific, as bone tenderness, swelling, fever, and leukocytosis may occur in both acute infarction and acute osteomyelitis. Blood cultures do not always yield a microbiological diagnosis, further hampering a precise diagnosis.

In this case, based on the presence of a subperiosteal collection, adjacent soft tissue extension, and the rapid recovery following antibiotic therapy, the final diagnosis of osteomyelitis was made.

Osteomyelitis in SCD arises from hematogenous spread of bacteria, facilitated by impaired splenic function, vascular stasis, and bone infarction, creating a nidus for infection [1].

CR rarely contributes to the diagnosis of acute complications of SCD, and MRI is recommended in any case of suspected acute MSK complications.
